# Tyrosine Kinase Inhibitors as Risk Factors for Cerebral Vascular Disease: Report of Two Cases and Literature Review

**DOI:** 10.1155/crnm/1871606

**Published:** 2025-12-20

**Authors:** Evangelos Papatolis, Stefania Kalampokini, Eleni Liouta, Stefanos Foinitsis, Evdoxia Hatjiharissi, Olga Kourti, Thomas Tegos, Marianthi Arnaoutoglou, Vasilios K. Kimiskidis

**Affiliations:** ^1^ 1st Department of Neurology, AHEPA University General Hospital, Thessaloniki, Greece; ^2^ Department of Radiology, AHEPA University General Hospital, Thessaloniki, Greece; ^3^ Hematology Unit, 1st Department of Internal Medicine, AHEPA University General Hospital, Thessaloniki, Greece; ^4^ Department of Cardiology, Laiko General Hospital, Athens, Greece, laiko.gr

**Keywords:** bosutinib, chronic myeloid leukemia, imatinib, nilotinib, stroke, tyrosine kinase inhibitors

## Abstract

Treatment for chronic myeloid leukemia (CML) with tyrosine kinase inhibitors (TKIs), especially nilotinib and ponatinib, has been associated with atheromatic vascular adverse events including cerebrovascular disease. Herein, we present two patients with CML and long‐term nilotinib treatment, who developed severe carotid atherosclerotic stenoses, both extra‐ and intracranial, resulting in ischemic stroke. The clinical and radiological findings as well as the possible pathophysiological mechanisms of these clinically significant complications are discussed. It seems that new‐generation TKIs such as nilotinib, ponatinib, and, to a far lesser extent, bosutinib increase the incidence of vascular occlusive events compared to imatinib, in a dose‐ and duration‐dependent manner. The mechanisms leading to vasculopathy are various and comprise promoting a prothrombotic platelet state, the dysregulation of glucose and lipid metabolism, increase of inflammatory cytokines, and affecting vessel wall endothelial cells. Regarding the outcome of cerebrovascular events, it seems that the discontinuation of TKIs alone or switching to a safer one is insufficient to resolve the stenoses of the cerebral arteries, even under dual antiplatelet treatment, anticoagulation, or high‐potency statin therapy. Thus, revascularization strategies such as extracranial to intracranial bypass surgery or stenting should be considered, especially when there is no improvement with medical treatment. These observations expand our knowledge on the association between TKIs and cerebral vascular disease, as well as provide more insights into the underlying pathogenesis. TKIs should not only be selected based on disease‐related variables but also based on patient‐related factors such as cardiovascular comorbidities.

## 1. Introduction

Chronic myeloid leukemia (CML) is a myeloproliferative disorder associated with the Philadelphia (Ph+) chromosome (generated by a reciprocal translocation between Chromosomes 9 and 22) and its oncogene *BCR::ABL1*, encoding a constitutively active tyrosine kinase that is found in > 90% of CML patients [1, 2]. Tyrosine kinase inhibitors (TKIs) prevent the launch of this pathway. Their use significantly improved the prognosis, response rate, overall survival, and patient outcome in CML patients [1, 2]. However, the remarkable response rate to imatinib in patients with CML was restricted by the incidence of resistant variants in the ABL kinase domain and molecular residual disease [1, 2].

The second‐generation *BCR::ABL1* TKIs, nilotinib, dasatinib, and bosutinib, were introduced shortly after, showing favorable outcomes in clinical trials in the majority of patients intolerant or resistant to imatinib therapy [1, 2]. However, emerging evidence of adverse effects has reached the spotlight, suggesting a linkage between long‐term *BCR::ABL1* TKI therapy, especially nilotinib, and vascular diseases, even though no confirmed pharmacological and pathophysiological mechanisms have been proven to justify this association [3–5]. The increased incidence of vascular occlusive events with TKIs became apparent retrospectively after second‐generation TKIs, particularly nilotinib, became commonly used in clinical practice, due to the high frequency of peripheral arterial occlusive disease [6]. In fact, a relatively high number of cerebrovascular disease cases associated with nilotinib have been reported, particularly from Asia [3, 7, 8], as well as in patients receiving ponatinib [9–12].

Herein, we report two cases of extensive multifocal intracranial (IC) stenoses, leading to ischemic stroke in two patients receiving long‐term treatment with nilotinib after previous treatment with imatinib. Interestingly, 3 months poststroke, and while the one patient was on bosutinib treatment, a follow‐up magnetic resonance angiography (MRA) showed further deterioration of the IC stenoses. Moreover, we did a literature search of all previously reported cerebrovascular events associated with the use of TKIs, nilotinib, bosutinib, and imatinib.

## 2. Case Presentation

### 2.1. Case 1

A 50‐year‐old man presented with left‐sided lower facial palsy and dysarthria upon awakening. The neurological examination revealed, additionally, a clumsy left hand, a left‐sided pronator drift, and mild gait difficulty (NIHSS Score 3). A brain computer tomography (CT) revealed a hypodense right frontal lesion, confirming the ischemic stroke. However, since the patient’s symptoms were present for longer than 24 h, thrombolysis and thrombectomy were not performed, and the patient continued the dual antiplatelet therapy (aspirin/ticagrelor) he was already receiving.

His past medical history comprised *BCR::ABL*‐positive CML, diagnosed 11 years before, and a myocardial infarction (MI) 2 months before the ischemic stroke, which was treated with percutaneous coronary angioplasty. CML had been initially treated with imatinib for 4 years with good response (MMR, ≤ 0.1% *BCR::ABL1* on the *BCR::ABL1* International Scale) and then changed to nilotinib 200 mg twice daily for 7 years with deep molecular response (MR^4^ ≤ 0.01% *BCR::ABL1*, MR^4,5^ ≤ 0.0032% *BCR::ABL1* on the *BCR::ABL1* International Scale), which was discontinued and replaced by bosutinib due to acute MI. Notably, predisposing vascular risk factors (e.g., smoking, hyperlipidemia, diabetes, and obesity) and a family history of cardiovascular and cerebrovascular disease were absent.

**Figure 1:**
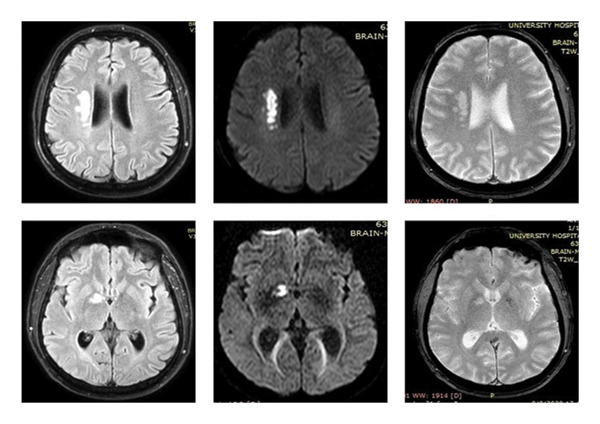
Brain MRI of Patient 1 in FLAIR, DWI, and T2 axial sections, respectively. The upper row shows a subacute ischemic stroke close to the right lateral ventricle with increased signal in all the above sequences. The lower row depicts a similar lesion of the right lenticular nucleus.

**Figure 2:**
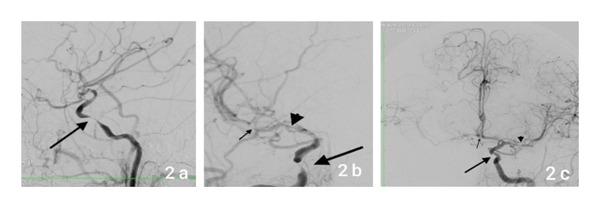
Digital subtraction angiography (DSA) of the right internal carotid artery (RICA) of Patient 1 (2a lateral and 2b anteroposterior projections, respectively) shows a severe stenosis of the cavernous RICA (large arrow), moderate stenosis of the right M1 segment (large arrowhead), and moderate stenosis of the right M2 segment (small arrow). DSA of the left internal carotid artery (LICA) (2c, anteroposterior projection) shows a moderate stenosis of the cavernous LICA (large arrow) and moderate stenosis of the left M1 (large arrowhead) with collateral circulation through the anterior communicating artery to the contralateral hemisphere (small arrow).

A multimodal neuroimaging approach with brain MRI–MRA (Figure 1) revealed the occlusion of the right internal carotid artery (ICA) and stenotic lesions in the right anterior (Α1 segment) and middle (Μ1 segment) cerebral artery. These findings were confirmed by digital subtraction angiography (DSA), which showed a significant stenosis of the cavernous segment of the right ICA (Figures 2(a), 2(b)), and moderate and severe stenoses of the right M1 and M2 branches, respectively (Figure 2(b)). In addition, DSA disclosed the moderate stenosis of the cavernous segment of the left ICA and minor stenosis in the peripheral left M1 branch (Figure 2(c)). Collateral flow through the anterior communicating artery was observed.

Cardiac embolism due to atrial fibrillation and valvulopathy was excluded by 24‐h Holter monitoring, while the transthoracic echocardiogram and transesophageal echocardiogram were normal. Laboratory testing for coagulopathy, systemic vasculitis, and rheumatic disease with vascular involvement was unremarkable. The patient was discharged with moderate improvement of dysarthria. It was recommended to continue dual antiplatelet therapy, with the addition of low‐molecular‐weight heparin in prophylactic dosage, along with a high‐potency statin and antihypertensive medications (angiotensin‐converting enzyme inhibitor and b‐blocker). He was also advised to continue bosutinib therapy for CML, according to hematological evaluation. However, 3 months poststroke, MRA revealed the deterioration of the IC stenoses (Figure 3), and since the patient’s CML was in remission (MR^4,5^ on the *BCR::ABL1* International Scale), the total cessation of TKI treatment was decided after hematological assessment. A follow‐up MRA, 3 months after bosutinib discontinuation, showed no further deterioration of the lesions. Meanwhile, 3 years after the ischemic stroke, treatment with antiplatelet and prophylactic low‐molecular‐weight heparin was switched to apixaban. MRA yearly in the following 5 years after the ischemic stroke showed stable angiographic findings. CML remained in deep molecular response (MR^4,5^ on the *BCR::ABL1* International Scale).

Figure 3Brain MRA of Patient 1 during hospitalization (a) and 3 months later (b). While the patient was receiving bosutinib, there was further worsening of the stenoses and flow at the level of the ICA (single arrow) and middle cerebral artery (MCA) (double arrow) on the right at three‐month poststroke MRA. After the total cessation of the TKI, no further deterioration was observed at 6‐month poststroke MRA.

**Figure:**
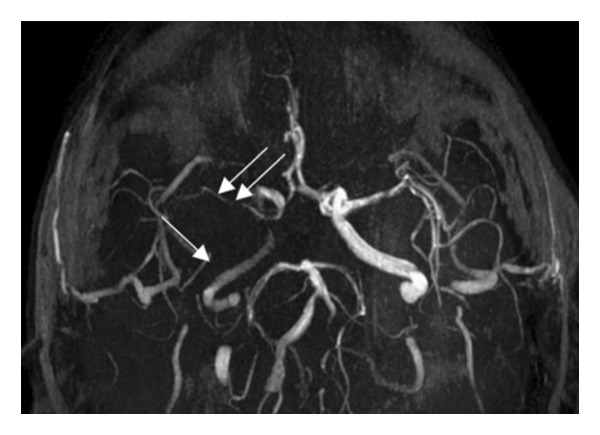
(a)

**Figure:**
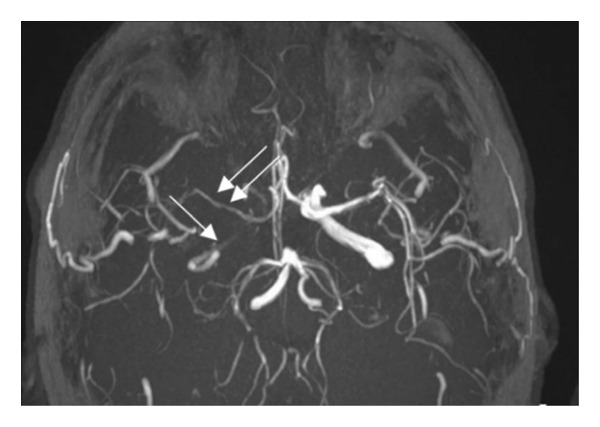
(b)

### 2.2. Case 2

A 70‐year‐old woman presented to our neurological department due to the numbness and weakness of her left hand upon awakening. On neurological examination, left central facial palsy, numbness of the left side of the tongue and cheek, left inferior quadrantanopia, and MRC 3/5 paresis of the left upper extremity, affecting mostly the finger flexors and extensors, were noted. Her past medical history was remarkable for Parkinson’s disease, thyroid cancer treated with thyroidectomy and radioactive iodine, and chronic phase, *BCR::ABL*‐positive CML in the last 15 years. The patient had received imatinib 400 mg daily in the first 8 years after CML diagnosis, with deep molecular remission (*BCR::ABL/ABL* 0.5% International Scale MR^4^, MR^4,5^), which had to be discontinued due to recurrent episodes of hemorrhagic eyelid edema and moderate anemia associated with the drug. Within 3 months after the discontinuation of imatinib, the patient developed a molecular recurrence and was switched to nilotinib 600 mg daily. She continued nilotinib for 5 years and remained in profound molecular remission (MR^4,5^ on the *BCR::ABL1* 0.5% International Scale)**,** until she developed parkinsonism, which was initially thought to be drug‐related, i.e., attributed to nilotinib. Nilotinib was stopped; nonetheless, molecular relapse occurred, and since the patient had a positive DaT‐scan with good levodopa response, nilotinib was restarted a few months later until presentation to our department.

Brain MRI revealed a subacute ischemic stroke in the cortical and subcortical areas of the right frontal lobe and another in the right occipital cortex. Time‐of‐flight (TOF) MR angiography revealed the subtotal occlusion of the cavernous segment of the right ICA, while its petrous segment and media cerebral artery MCA were sufficiently depicted (Figure 4). There was also a paucity of the peripheral vascular branches of the right MCA in the frontal and parietal areas. DSA revealed a moderate to severe stenosis of the right ICA just above the carotid bifurcation with an ulcerated atherosclerosis plaque. Τhere was also subtotal occlusion of the right ICA at the genu and significant stenoses of M2 and A1 segments on the right (Figure 5). There was also a moderate stenosis of the left ICA above the carotid bifurcation and significant stenosis of its supraclinoid segment. Lastly, the patient had a moderate stenosis of the V4 segment of the left vertebral artery and the left posterior cerebral artery (PCA) after P1 segment and occlusion of the right PCA after P1 segment.

Figure 4DWI sequence of brain MRI of Patient 2 showing subacute ischemic stroke in the cortical and subcortical area of the right frontal lobe (a) and another in the right occipital cortex (b). MRA showing the subtotal occlusion of the cavernous segment of the right ICA (arrow) (c).

**Figure:**
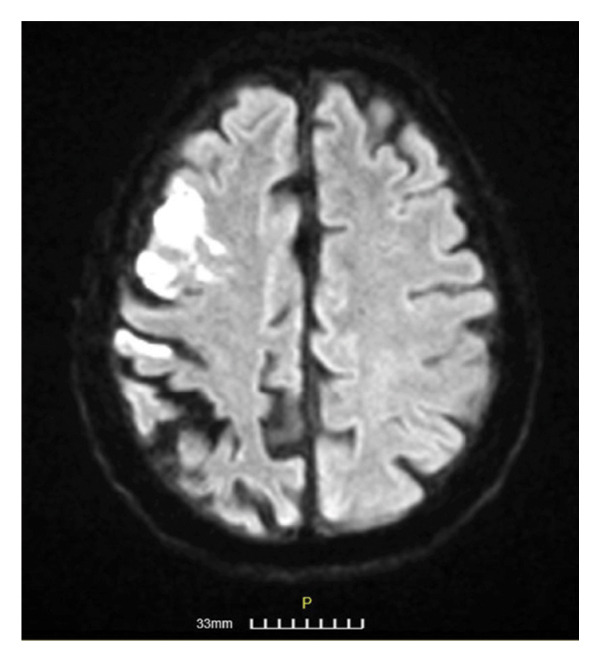
(a)

**Figure:**
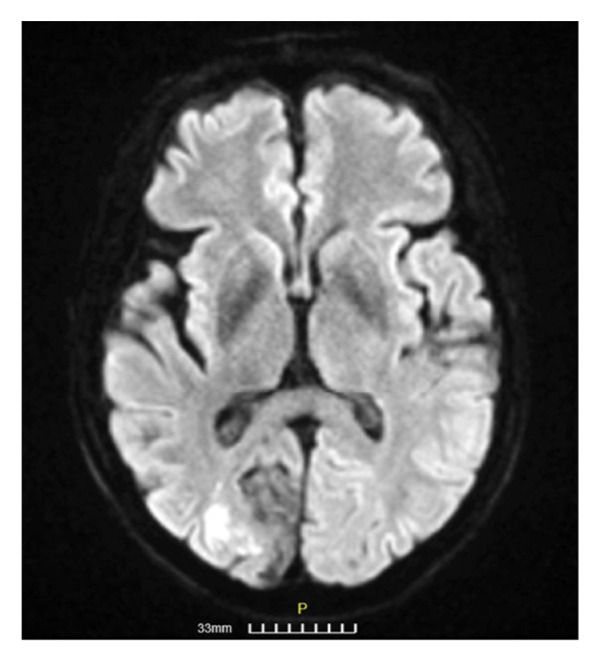
(b)

**Figure:**
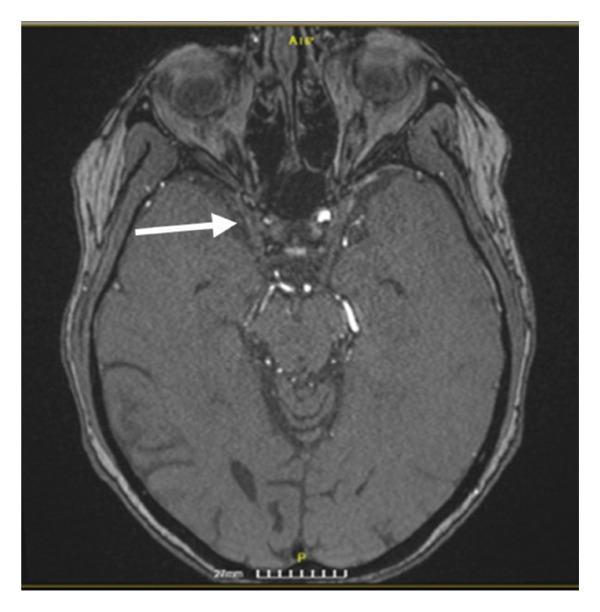
(c)

Figure 5Digital subtraction angiography (DSA) reveals a moderate to severe stenosis of the right ICA above the carotid bifurcation (a), subtotal occlusion of the right ICA at the genu (b), and significant stenoses of M2 (white arrow) and A1 (black arrow) on the right (c).

**Figure:**
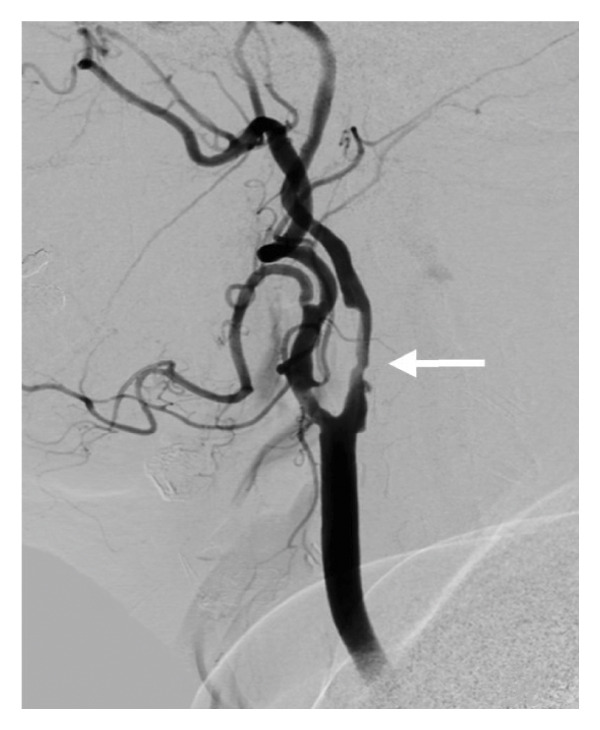
(a)

**Figure:**
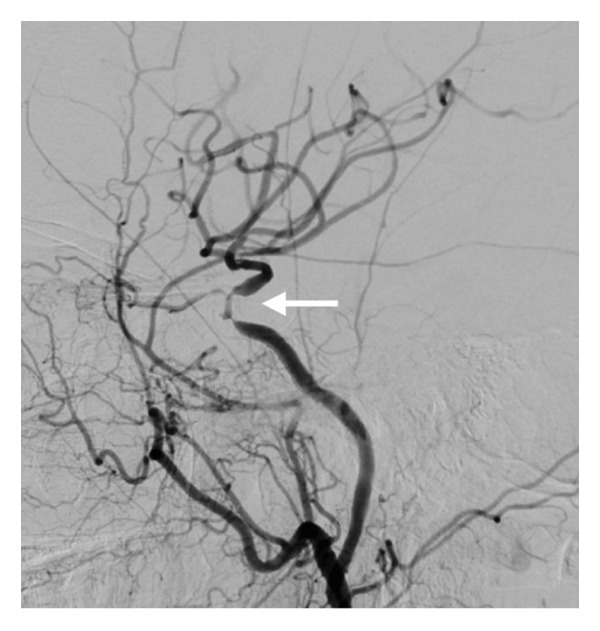
(b)

**Figure:**
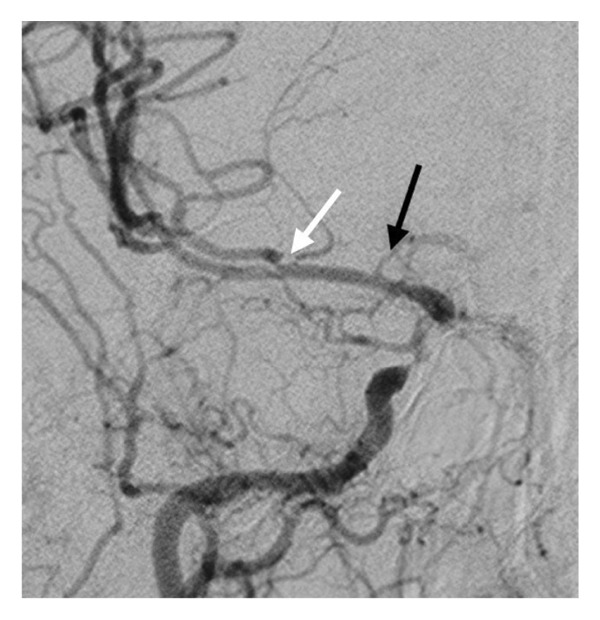
(c)

Predisposing vascular risk factors and family history of cardiovascular disease were absent. IL‐6 in serum was measured and was found to be increased (10.2 pg/mL, reference values < 5.7). The rest laboratory testing for systemic and infectious vasculitides or rheumatic diseases was unremarkable. The patient was treated with dual antiplatelet and high‐dose statin therapy. Nilotinib was permanently discontinued, and the patient remained in deep molecular response in the following 6 months (MR^4,5^ on the *BCR::ABL1* International Scale). Six‐month‐follow‐up by MRA showed stable findings.

Vascular events in relation to CML course in the two cases can be seen in Table 1.

**Table 1:** Vascular events in relation to chronic myeloid leukemia course in the two cases.

	Patient’s demographics (at presentation)	Past medical history	CML therapies	Duration	Molecular response	Vascular events/outcome
Case 1	Male, 50 years old	CML	Imatinib	2009–2013	MMR	None
			Nilotinib	2013–2020	MR^4^, MR^4,5^	Myocardial infarction, ischemic stroke 2020
			Bosutinib	2020 (for 3 months)	MR^4,5^	Deterioration of intracranial stenoses
			None	2021–present	MR^4^, MR^4,5^	None, stable intracranial stenoses under NOACS and high‐potency statin
Case 2	Female, 70 years old	CML, Parkinson’s disease, thyroid cancer	Imatinib	2010–2018	MR^4,5^	None
			Nilotinib	2018–spring 2025	MR^4,5^	Ischemic stroke 2025
			None	Spring–fall 2025	MR^4,5^, MR^4^	None, stable intracranial stenoses under dual antiplatelet and high‐potency statin therapy

Abbreviations: CML: chronic myeloid leukemia, MMR ≤ 0.1% *BCR::ABL1*, MR^4^ ≤ 0.01% BCR::ABL1, MR^4,5^ ≤ 0.0032% on the *BCR::ABL1* International Scale (IS), NOACs: novel oral anticoagulant drugs.

## 3. Discussion

Our patients’ multifocal vascular disease, which comprised ischemic heart disease and ischemic cerebrovascular events due to bilateral cerebrovascular stenoses, was most likely associated with the long‐term nilotinib treatment for CML. Cerebrovascular ischemic events due to cerebral vessel stenoses associated with nilotinib have been reported after a wide range of treatment durations, ranging from 7 days to 12 years [13, 14]. Most of the previously reported cases of stenoses affecting cerebral arteries due to TKIs, in particular nilotinib and ponatinib, involve more often the IC than the extracranial (EC) parts of the vessels, with the carotid system being more commonly affected than the vertebrobasilar system [5, 13, 15–23]. Moreover, our second patient was previously treated with imatinib. Cerebrovascular events in patients with CML previously treated with imatinib have been reported in association with subsequent treatment with other TKIs such as nilotinib and dasatinib [15, 19, 21, 24–26]. The fact that the cerebral infarction in our first patient occurred while he was being treated with bosutinib for the previous 3 months, as well as the further worsening of the IC stenoses in follow‐up MRA, may also implicate bosutinib in the occurrence of vascular adverse events. Of note, cases of aggravation of IC stenosis despite switch to bosutinib are rare [15, 27]. All previously reported cases of cerebrovascular events due to the stenoses of cerebral vessels can be seen in Supporting Table 1.

An extensive clinical and laboratory evaluation did not provide evidence for alternative diagnoses, such as systemic vasculitides, rheumatic diseases with vascular involvement as well as infectious causes with vascular complications. Finally, the systemic (coronary) involvement in the first patient made primary CNS vasculitis unlikely. In most CML patients under nilotinib where recurrent vascular events were reported, with or without previous treatment with another TKI (most commonly imatinib), one or more risk factors for the evolution of atherosclerosis were identified [5, 13, 15–17, 21, 23, 28–30]. On the other hand, cases of ischemic stroke without any concomitant vascular risk factor have also been previously reported [14, 18, 19, 27, 31–33], even in patients younger than 40 years old [20].

Regarding the outcome of cerebrovascular events, it seems that the discontinuation of TKIs alone or switching to a safer one such as dasatinib is insufficient to resolve the stenoses of the cerebral arteries, even under dual antiplatelet treatment or anticoagulation [18, 19, 22, 23, 25, 27, 32]. Thus, revascularization strategies such as EC to IC bypass surgery or stenting should be considered, especially when there is no improvement with medical treatment, i.e., antiplatelet and high‐potency statin treatment [13]. Outcomes were favorable in most cases receiving revascularization [13, 17, 20, 26], although follow‐up longer than 1 year is not available. On the other hand, re‐stenosis after IC stent placement has also been reported [15].

Since the introduction and approval of TKIs as front‐line therapy in CML, there has been a remarkable improvement regarding the prognosis of CML patients [1]. Most patients receiving TKI treatment will be advised to continue this therapy indefinitely, while 20%–40% will be able to discontinue it after achieving a deep molecular remission [1, 34, 35]. Nevertheless, long‐term administration of TKIs, especially nilotinib and ponatinib, has been associated with severe vascular adverse events, including cerebrovascular disease, peripheral arterial disease, coronary artery disease, and carotid and renal artery stenosis [1, 5, 10–12, 36]. According to clinical data, the new‐generation *BCR::ABL1* TKIs (in particular nilotinib and ponatinib) increase the incidence of vascular occlusive events compared to imatinib or dasatinib, in a dose‐ and duration‐dependent manner, especially in patients with higher baseline Framingham cardiovascular risk scores [31, 37]. On the other hand, many studies have shown that it is possible for CML patients who achieve a long‐term deep molecular response to stop TKI treatment and maintain remission [38]. In this treatment‐free remission (TFR), the long‐term adverse events of TKIs, such as cerebrovascular events, can be mitigated; however, the decision of discontinuing TKIs should be based on strict patient selection, taking into account factors such as age, immune profile, and measurable residual disease levels [39].

The mechanisms leading to vasculopathy have not yet been clarified, although possible scenarios have been suggested. Nilotinib promotes a prothrombotic platelet state, with an increase in protease‐activated receptor‐1 (PAR‐1)–mediated platelet secretion, adhesion, and activation, as well as an increase in the release of adhesive molecules, without impairing platelet aggregation [4, 40]. In contrast, other TKIs, such as dasatinib and imatinib, are more likely to cause hemorrhagic events via an unknown molecular mechanism of platelet dysfunction [4, 41–43]. Moreover, nilotinib has been associated with glucose metabolism dysregulation, as it raises blood glucose levels, leading to overt diabetes mellitus in many cases [4, 44]. Insulin resistance is the leading theory for how hyperglycemia develops in those cases [4, 45]. Occasionally, there is reduced insulin secretion, most likely as a result of pancreas β‐cell depletion [4]. There is little information about how bosutinib affects glucose metabolism and there have not been any reports of significant changes in glucose profile under bosutinib [4, 45]. Regarding lipid metabolism, an increase in cholesterol levels is linked to nilotinib [44]. This rise in lipid synthesis might be caused by insulin resistance [4, 46] and/or the dysregulation of low‐density lipoprotein receptor (LDLR) and lipoprotein lipase (LPL), thus reducing blood lipid clearance [47]. However, there is little evidence that proper management of lipid metabolism in patients receiving *BCR::ABL1* TKIs will stop arterial thrombosis and prevent vascular occlusive events [46, 48]. On the other hand, bosutinib and imatinib do not seem to affect lipid metabolism [49, 50].

Nilotinib also decreases endothelial cell proliferation and thus inhibits the regeneration of endothelium; it also prevents the migration of endothelial cells disrupting angiogenesis [4]. Nilotinib was also found to increase proatherogenic adhesion proteins on endothelial cells, e.g., Intercellular Adhesion Molecule 1 (ICAM1), Vascular Cell Adhesion Protein 1 (VCAM1), and E‐selectin, which induce vascular events by recruiting inflammatory cells and platelets [4, 51]. Notably, bosutinib, unlike nilotinib, does not interact with several clinically relevant targets such as platelet‐derived growth factor receptor (PDGFR) [52]. In addition, some TKIs, including nilotinib, increase plasma levels of inflammatory cytokines Interleukin‐6 and Inteleukin‐1‐beta and/or high‐sensitivity C‐reactive protein, which can lead to atherosclerotic endothelial changes [53–55]. Indeed, our second case had increased serum levels of Interleukin‐6, indicative of an inflammatory process. It is believed that in patients receiving nilotinib, an induced “inflammatory/oxidative status,” together with a genetic pro‐atherothrombotic predisposition (such as carrying the G allele of LOX‐1 polymorphism), may favor the increased incidence of cardiovascular events [56]. In fact, patients carrying the RNF213 p.4810K variant treated with nilotinib might be more susceptible in developing IC arterial stenosis or occlusion, mostly reported in Asian patients [23].

In conclusion, we present two cases of severe IC vascular stenoses, resulting in ischemic stroke, in patients who previously received long‐term treatment with nilotinib. The first one received bosutinib at stroke occurrence with further deterioration of the stenoses in the follow‐up, while both had a previous long‐term treatment with imatinib. It is worth mentioning that no vascular risk factors existed for both patients. These data further expand the incidence of cerebrovascular adverse events due to bosutinib administration, which is considered safer regarding cerebrovascular events, compared to other TKIs and especially nilotinib.

Future well‐designed studies can expand our knowledge on the association between TKIs and vascular disease, as well as provide more insight into the underlying pathogenesis. Taking into account the patient’s cardiovascular comorbidities is of utmost importance to select the TKI therapy with the most favorable vascular adverse event profile. Therefore, TKIs should not only be selected based on disease‐related variables like *BCR::ABL1* mutations but also be based on patient‐related factors, i.e., it should be personalized [57]. On the other hand, the risk of developing severe vascular adverse events under TKI therapy can be decreased by lowering the dose, shortening the time of exposure, or even switching to another safer TKI for a short period, whenever possible [57]. Another consideration would be to start a prophylactic treatment with antiplatelets in patients under long‐term treatment with TKIs, after weighing their vascular risk factors or family history. Neurologists, oncologists, cardiologists, and radiologists must be informed of these issues and cooperate to design effective monitoring and risk mitigation strategies.

## Consent

Written informed consent has been obtained from the patients to publish their cases in this paper.

## Disclosure

All authors have read and agreed to the published version of the manuscript.

## Conflicts of Interest

The authors declare no conflicts of interest.

## Author Contributions

Conceptualization and methodology: E.P., E.L., and M.A; software: E.P. and S.F.; validation: V.K.K., M.A., T.T., and S.F.; formal analysis: E.P., E.L., and S.K; investigation: S.K., E.P., and E.L.; resources: S.K, E.P., E.L., and E.H; data curation: E.P., E.H, S.K; writing–original draft preparation: E.P., E.L., S.K, and M.A.; writing–review and editing: E.P., E.L, Ε.Η., and T.T.; visualization: S.F.; supervision: M.A., T.T., and V.K.K.; and project administration: M.A. and V.K.K.

## Funding

This research did not receive a specific grant from any funding agency in the public, commercial, or not‐for‐profit sectors.

## Supporting Information

Supporting Table 1. Tyrosine kinase inhibitors (imatinib, nilotinib, and bosutinib) associated with cerebral vascular events.

## Supporting information


**Supporting Information** Additional supporting information can be found online in the Supporting Information section.

## Data Availability

The data that support the findings of this study are available from the corresponding author upon reasonable request.
