# Supplementary material for: Tyrosine Kinase Inhibitors as Risk Factors for Cerebral Vascular Disease: Report of Two Cases and Literature Review
Source: Case Rep Neurol Med. 2025 Dec 20;2025:1871606. doi: 10.1155/crnm/1871606 (PMC12747102; doi:10.1155/crnm/1871606)
Supplement: Supplementary file 1 — Supporting Information Additional supporting information can be found online in the Supporting Information section. [file CRNM-2025-1871606-s001.docx]

| Author et al, year | No of patients | Sex (F/M) | Age | TKI treatment | Concomitant risk factors | Cerebral vascular events | Cerebral arterial stenosis | Treatment | Outcomes |
| --- | --- | --- | --- | --- | --- | --- | --- | --- | --- |
| Alshiekh-Nasany et al, 2016 (19) | n=1 | M | 50 | - Imatinib for 4 years - nilotinib for unspecified period | - Mildly elevated LDL cholesterol elevated | - two transient episodes of right arm and leg weakness and numbness - dysarthria | - severely narrowed bilateral MCA, with patent neck arteries (“puff of smoke” appearance | - antiplatelet therapy - high-potency statin - nilotinib was replaced with another TKI - Bilateral direct revascularization | - The patient remained symptom-free at 6-month follow-up |
| Chen et al, 2018 (8) | n=1 | F | 49 | - Imatinib for 6 years - nilotinib for unspecified period | - Type 2 diabetes mellitus - hyperlipidemia | - episodes of transient right-sided weakness - paresthesia - speech difficulties - TIA symptoms | - severe multifocal stenoses in the left ICA | - nilotinib was switched to bosutinib - placement of stent in her right coronary artery - high intensity statin and dual antiplatelet therapy | - Improved distal blood flow with intracranial stent placement in ICA. - One month following stent placement, right-sided numbness with re-stenosis distal and proximal to the stent construct. - dual antiplatelet therapy continued, 3- month follow-up angiogram revealed near occlusion of intracranial segments of left ICA |
| Coon et al, 2013 (5) | n=1 | F | 70 | - Imatinib for 4 years - Tipifarnib was added - nilotinib for unspecified period | *During nilotinib therapy*   - hypertension - hyperlipidemia - prediabetes | *During Imatinib therapy*   - left-sided weakness - dysarthria | - multifocal narrowing in MCA and PCA bilaterally - 50–60% narrowing of the right ICA | - imatinib was switched to nilotinib - aspirin, clopidogrel, and warfarin | - Under nilotinib treatment right MCA distribution ischemic stroke with high-grade stenosis (95%) of the extracranial right ICA. - Due to high risk of surgery, maintained on dual antiplatelet therapy and anticoagulation |
|  |  |  |  |  |  | *During nilotinib therapy*   - confusion - weakness and sensory symptoms of left upper extremity |  |  |  |
| Gómez-Galván et al, 2017 (9) | n=3 | M | - Pt1:66 - Pt2: 56 - Pt3:66 | nilotinib   - Pt1: 8 months - Pt2: ~4 years   Pt3: 7 years | - Pt1: arterial hypertension - Pt2: smoking, arterial hypertension and coronary artery disease - Pt3: NR | - Pt1: vertigo, diplopia, central facial palsy, gait ataxia, due to multiple ischemic lesions in the brainstem and occipital cortex - Pt2: dysarthria, hemiparesis, and hemihypaesthesia - Pt3: transient episodes of hemiparesis and left hemihypaesthesia, due to multiple ischemic lesions in the right frontal and parietal cortex | - Pt1: vertebral artery occlusion and significant intracranial atherosclerosis - Pt2: central retinal artery occlusion, near occlusion of left ICA and stenosis of right ICA and both MCA - Pt3: ICA dissection and MCA stenosis (more than 50%) | - Pt1: nilotinib switched to dasatinib and oral anticoagulant acenocoumarol added - Pt2: anticoagulant treatment with intravenous sodium heparin - Pt3: nilotinib suspended, treatment with lipid-lowering drugs and anticoagulant agents initiated | - Pt1: n/a - Pt2: Despite treatment, hemiplegia and aphasia due to left ICA occlusion. Emergency angioplasty and stent placement no benefit; stent occlusion shortly after. The patient remained on anticoagulant, which was later-on replaced with aspirin - Pt3: At 3-month follow-up ICA occlusion persisted and acenocoumarol was replaced with aspirin. |
| Hirayama et al, 2022 (10) | n=2 | F | - Pt1:46 - Pt2: 43 | - Pt2: imatinib for 16 years   nilotinib   - Pt1: 10 years   ponatinib   - Pt2: 3 months | - Pt1: smoking history, high level of LDL-cholesterol - Pt2: smoking history | - Pt1: NR - Pt2: chronic headache, dizziness | - Pt1: severe left ICA stenosis and moderate right ICA stenosis - Pt2: multiple stenosis in the bilateral intracranial ICAs and the right PCA | - Pt1: aspirin, clopidrogel and statins before stent placement - Pt2: antiplatelet agents | Pt1: Stenosis was reduced to 10%. Nilotinib was continued; no changes in bilateral ICA lesions in 3 months following stent placement.   - Pt2: Partial improvement in bilateral ICAs and PCA. Ponatinib continued; no new neurological event occurred in 3- month follow-up. |
| Jager et al, 2014 (18) | n=1 | F | 69 | - Imatinib for 14 years - nilotinib for 7 months | - none | - aphasia | - n/a | - acetylsalicylic acid, clopidogrel, simvastatin - amlodipine and bisoprolol | - Second severe stroke 3 months later. Acetylsalicylic acid was replaced with acenocoumarol. - Few months later, despite adequate anticoagulation, third ischemic stroke. |
| Kakadia et al, 2021 (11) | n=1 | M | 55 | nilotinib for 2 years | - none | - aphasia - dysarthria - right hemiparesis - acute infarction in the left hemisphere | - high grade stenosis of contralateral MCA - severe stenosis of P2 segment of right PCA | - intravenous alteplase - mechanical thrombectomy of left MCA with recanalization and subsequent re-occlusion - discontinuation of nilotinib - aspirin and clopidrogel | - Right MCA stenosis improved 6 months later. However, stenosis of left M1 and right P2 were persistent. - No further clinical or radiographic vascular events were observed 11 months later. |
| Ozaki et al, 2017 (12) | n=1 | M | 74 | - imatinib for 3,5 years - nilotinib for 2,5 years | - none | - Repeated TIA (transient hemiplegia) | - narrowing of left intracranial ICA and basilar artery | - discontinuation of nilotinib - switch to bosutinib, but discontinued due to hepatic function impairment - switch to dasatinib, but discontinued due to repeated TIA | - Dual antiplatelet therapy unable to control TIA. - Percutaneous transluminal angioplasty in the stenotic right ICA followed by intracranial stent placement. - Successful cerebral vascular revascularization with no further TIAs. - bosutinib treatment resumed; patency of ICA confirmed three months later |
| Rai et al, 2023 (13) | n=1 | F | 39 | - imatinib - dasatinib - ponatinib - nilotinib for 3,5 years | - increased LDL levels | - repeated transient weakness of left extremities due to acute and subacute infractions | - severe stenosis in right MCA, right ACA, right posterior cerebral artery, right vertebral artery and left common carotid artery | - dual antiplatelet therapy - discontinuation of nilotinib - revascularization surgery | - Neither neurological deficits nor new cerebral infarction were observed - good bypass patency for at least 6 months |
| Sibal et al, 2024 (14) | n=1 | M | 49 | - Imatinib - nilotinib for 7,5 years | - increased LDL levels | - right-ataxic hemiparesis, motor and sensory deficits - imbalance and right-sided weakness - blurred vision in the right eye - dysarthria | - hypoperfusion in the left occipitotemporal lobe and MCA - focal occlusion of left PCA - focal narrowing of left supraclinoid ICA, right supraclinoid ICA, right MCA - mild narrowing of the left M1 segment of MCA | - dual antiplatelet therapy - atorvastatin - switch back to imatinib | - Two months follow-up improved symptoms, outcomes on stenoses n/a. |
| Suzuki et al, 2018 (15) | n=1 | M | 55 | nilotinib for 3 years | - mildly elevated LDL levels - occasional consumption of alcohol - sporadic smoking | - mild left‑sided hemiparesis - dysarthria - dysphagia | - acute cerebral infarction in the right MCA - severe stenosis of right ICA and left MCA - diffuse concentric thickening of the vessel wall of the right intracranial ICA and MCA, and the left MCA | - discontinuation of nilotinib - antiplatelet therapy - statins | - revascularization of the right MCA - thereafter, no recurrence of brain ischemia |
| Uemura et al, 2020 (16) | n=2 | M | - Pt1:62 - Pt2: 59 | nilotinib   - Pt1: 9 years   Pt2: 7,5 years | - Pt1: father’s history of cerebral infraction - Pt2: history of hypertension | - Pt1: dysesthesia of left upper limb, recurrent transient left hemiparesis - Pt2: gait disturbance, hoarseness, dysarthria, left hemiparesis | - Pt1: multiple occlusions of the intracranial arteries including the right MCA, the left ACA and the left PCA - Pt2: multiple occlusions of the intracranial artery including the left MCA and the basilar artery, acute infarcts in occipital lobe and cerebellum | - Pt1: switch to dasatinib - Pt2: antiplatelet therapy (aspirin and clopidrogel, argatroban) | - Pt1: patient uneventful for two years. - Pt2: Locked-in state due to infarction of midbrain and pons. |
| Sasaki et al, 2025 (6) | n=1 | M | 74 | nilotinib for 12 years | - diabetes mellitus | - gait disturbance | - 70% stenosis at left MCA at 6 years - severe bilateral intracranial ICA and MCA stenoses at 12 years - diffuse stenosis in M1 - 80% stenosis in the left ICA | - initially clopidogrel - bypass surgery and stent in the left ICA at 12 years - nilotinib discontinued | - one-year follow-up after bypass surgery: no symptom recurrence |
| Yoon et al, 2021 (21) | n=1 | M | 46 | - Imatinib - nilotinib for 8 years | - increased levels of glycated hemoglobin levels | - recurrent episodes of transient left-sided weakness | - bilateral stenosis of MCA | - clopidrogel - switch to dasatinib | - No progression of MCA stenosis at approximately two-year follow-up |
| Sim et al, 2021 (45) | n=1 | M | 51 | nilotinib for ~3.5 years | - hyperglycemia | - dysarthria | - stenosis of left MCA | - aspirin - atorvastatin - switch to dasatinib | - no recurrence of neurological symptoms at one-year follow-up |
| Corrêa et al, 2023 (24) | n=1 | M | 67 | nilotinib for unspecified period | - none | - left-sided paresis - acute ischemic lesions in the right frontal and parietal lobes | - stenosis of right MCA | - anticoagulants - statin - switch to imatinib | - n/a |
| Tokatlı et al, 2024 (25) | n=1 | F | 70 | - imatinib for 6 years - dasatinib for 6 years - nilotinib for 3 years | - none | - inter-arm blood pressure difference | - significant stenosis of left subclavian and vertebral arteries - severe stenosis of right ICA | - endovascular stent placement in left subclavian artery - dual antiplatelet therapy, statin | - One year later, progressive stenosis of right ICA and new stenosis of left proximal ICA - nilotinib switched to bosutinib |
| Agrawal et al, 2024 (26) | n=1 | F | 58 | nilotinib for ~1,5 year | - none | - right-sided hemiparesis - dysarthria | - stenosis in the left MCA | - dual antiplatelet therapy - atorvastatin | - n/a |
| Hersant et al, 2019 (20) | n=1 | M | 61 | - imatinib for 2 years - dasatinib for 2 years - nilotinib for ~3,5 year | - none | - hyperlipidemia - hypertension | - stenosis (10%-30%) of right ICA | - aspirin - ramipril - switch to bosutinib | - Aggravation of the right ICA to 70% - Endarterectomy, thereafter no significant re-stenosis |
| Quintás-Cardama et al, 2012 (7) | n=1 | F | 50 | nilotinib for 7 days | - none | - dysarthria - memory loss - poor concentration - confusion | - n/a | - discontinuation of nilotinib | - episode with dysarthria and disorientation few weeks after nilotinib treatment was resumed - nilotinib discontinued, imatinib initiated |
| Bramucci et al, 2024 (23) | n=1 | M | 55 | nilotinib for 6 years | - family history for cardiovascular disease | - asymptomatic patent foramen ovale | - significant left ICA stenosis | - dual antiplatelet therapy | - switched to bosutinib - carotid endarterectomy and dual antiplatelet therapy. At 2-month follow-up no restenosis |

**Abbreviations:** TKI: tyrosine-kinase inhibitor, ICA: internal carotid artery, MCA: middle cerebral artery, PCA: posterior cerebral artery, n/a: not reported

**Supplementary table 1. Tyrosine-kinase inhibitors (imatinib, nilotinib, bosutinib) associated cerebral vascular events**
